# Cannabinoid Receptors and Their Relationship With Chronic Pain: A Narrative Review

**DOI:** 10.7759/cureus.10436

**Published:** 2020-09-14

**Authors:** Adarsh Thomas Anthony, Shermeen Rahmat, Prerna Sangle, Osama Sandhu, Safeera Khan

**Affiliations:** 1 Internal Medicine, California Institute of Behavioral Neurosciences & Psychology, Fairfield, USA

**Keywords:** chronic pain management, cannabinoid receptors, cb1 receptor, cb2 receptor, inflammatory pain, antinociception, opiod alternative

## Abstract

The burden of chronic pain has affected many individuals leading to distress and discomfort, alongside numerous side effects with conventional therapeutic approaches. Cannabinoid receptors are naturally found in the human body and have long been an interest in antinociception. These include CB1 and CB2 receptors, which are promising candidates for the treatment of chronic inflammatory pain. The mechanism of action of the receptors and how they approach pain control in inflammatory conditions show that it can be an adjunctive approach towards controlling these symptoms. Numerous studies have shown how the targeted approach towards these receptors has activated them promoting a release in cytokines, all leading to anti-inflammatory effects and immune system regulation. Cannabinoid activation of glycine and gamma-aminobutyric acid (GABA) models also showed efficacy in pain management. Chronic conditions such as osteoarthritis were shown to also benefit from this considerable treatment. However, it is unclear how the cannabinoid system works in relation with the pain pathway. Therefore, in this review we aim to analyse the role of the cannabinoid system in chronic inflammatory pain.

## Introduction and background

Pain is the most sought out reason why Americans seek medical attention, and comprehensive literature has identified the relevance of the endocannabinoid pathway in controlling pain [1]. The activation of cannabinoid receptors is believed to have antinociceptive effects in controlling the human perception of pain. Inflammatory chronic pain conditions have been thought to be controlled through the cannabinoid receptors and affect symptomatic relief.

Studies performed in this interest have been shown to have positive outcomes. A noteworthy example was the rhesus monkey warm-water tail withdrawal paradigm. In this study, anandamide (endogenous cannabinoid) and WIN55 (cannabinoid receptor agonist) both showed dose-dependent antinociceptive effects [2]. This emphasizes the fact that the cannabinoid receptors affect the role of action in controlling this unpleasant sensation, known to man as pain. As a topic of interest, certain clinical studies prove that medicinal cannabis or cannabinoid-based medications relieve the burden of pain in chronic diseases such as fibromyalgia, multiple sclerosis, and even cancer [3]. These chronic pain conditions have all been very difficult conditions to treat, and many physicians have found success with cannabinoid-based treatment due to the positive outcome in their patients.

Several studies indicate that the cannabinoid system regulates nociceptive thresholds, raising the possibility that the inactivation and hypoactivity of the cannabinoid system prolong or produce hyperalgesia and chronic pain [4]. Activation of these receptors may play a role in the anti-inflammatory process of these chronic pain conditions. Considering the immunomodulatory effects of these drugs, it is also important to investigate their modulation in chronic inflammatory pain [5]. The resulting immune-cell migration and cytokine production show the endocannabinoid-mediated effect and its relation in the host inflammatory response [5].

There are two important cannabinoid receptors known as CB1 and CB2 found in the human body. CB1 receptors are expressed predominantly in the central nervous system (CNS), whereas CB2 receptors are found mostly outside the CNS [6]. These receptors, when activated, can suppress the pain stimulus through different mechanisms. Neurochemical, behavioral, and electrophysiological studies all demonstrated the modulation of inflammatory nociception through CB2 receptor activation [6]. The receptors have been investigated to see their role in a variety of pain states, including inflammatory and neuropathic pain conditions. 

The use of cannabinoid components and their psychomimetic actions or potential for abuse have dampened the enthusiasm for their therapeutic development. On the contrary, CB2 receptor-selective agonists have shown to reduce inflammation and pain, without eliciting the negative cannabinoid behavioral effects [7]. If a targeted approach is made to isolate the cannabinoid receptors, the effects on the treatment of chronic pain can be valuable. It is also important to note that it is unknown if the effect of these receptors acts through the immune system or has a direct action on the pain pathway; therefore, we aim to explore the effect of cannabinoid receptors on inflammatory and chronic pain.

## Review

The mechanism of action, the effect on the CB1 and CB2 receptors, and understanding of the antinociceptive effects of the cannabinoid receptors give insight into the understanding of its role in inflammatory pain. 

Mechanism of action

A variety of studies are aimed at determining the mechanism of action of the cannabinoid receptors. The endocannabinoid system mainly focuses on two key receptors known as CB1 and CB2. These receptors can be activated either directly or indirectly, and once these receptors are activated, it is key to determine the relation to pain [8]. Immune functional events have been widely linked to CB2 receptors [9]. In one study, it was proposed that the immune-mediated response from the activation of the cannabinoid receptors resulted in the release of anti-inflammatory mediators, including macrophages, interleukin (IL)-10, interferon (INF)-γ and IL-12 [5]. Another study showed regulation of cannabidiol, causing a reduction in mediators such as prostaglandin E [10]. This regulation is in the balance as it also regulates proinflammatory cytokine production [5]. The combination of immunosuppression leading to anti-inflammatory factors points towards a decrease in chronic inflammatory pain states. The overall findings showed that it reduced immune cell proliferation, activation, and apoptosis of these cells [3].

Another study discussed how the action of cannabinoids demonstrated analgesic and immunomodulatory changes in arthritis and other chronic diseases. Fibroblast-like synoviocytes (FLS) are cells that are located in synovial tissue. Patients affected by rheumatoid arthritis and osteoarthritis are shown to have FLS, which express the CB1 and CB2 receptors [11]. This can be inferred that the activation of the fibroblast synoviocytes can depress the pain stimulus in these chronic inflammatory states. Furthermore, regarding osteoarthritis, a study determined that both the CB1 and peroxisome proliferator-activated receptor (PPAR)-α were mediators in controlling pain [12]. This confirms that cannabinoid receptors affect and modulate inflammation through multiple pathways as an adjunctive aid in chronic inflammatory pain [3].

Bringing it down to a more molecular level, studies identified the action through cannabinoid receptors being activated via G(i/o) proteins, negatively modulate cyclic adenosine monophosphate (AMP) levels, and activated inward rectifying K(+) channels [13]. The G protein-coupled receptors, when activated, send signals downwards and may also have a role in the regulation of gamma-aminobutyric acid (GABA) and glutamate release [1]. Both these targets are known to affect the pain pathway. Glycine receptors also play a significant role as potentiation of the receptors showed analgesic properties in mice [14]. The potentiation of the cannabinoid glycine receptors may be contributing to the therapeutic effect of cannabinoids [15].

Fatty acid amide hydrolase (FAAH) is a major enzyme that degrades fatty acid amides and binds to cannabinoid receptors. Using FAAH in animal models, both CB1 and CB2 receptors were studied, it was interestingly noted that the CB2-expressing peripheral immune cells and glial cells of the CNS played a role in models of chronic inflammatory pain relief [16]. The body regulates and controls the number of circulating endocannabinoids, which are dynamically involved in pain signaling [17].

Opioid use for pain has been a widely problematic approach to pain management with the risks and side effects, but with co-administration of cannabinoid agonists, symptoms were found to improve. This was studied and determined that when the CB2 agonist was administered with morphine, it synergistically affected the inflammatory pain response in a dose- and time-dependent matter [18]. This finding proved that opioid use in conjunction with CB2 agonists led to fewer side effects, all while maintaining adequate pain control.

CB1 versus CB2

**Figure 1:**
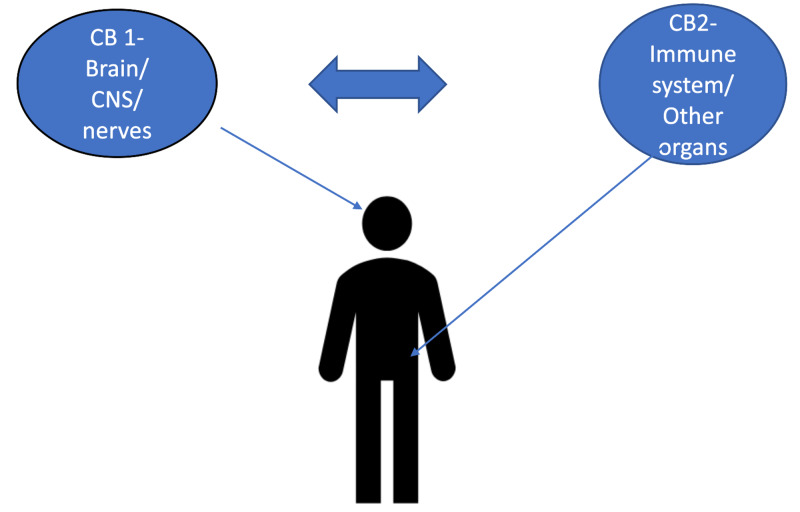
CB1 versus CB2 receptors CNS, central nervous system

There are two major cannabinoid receptors targeted by the endocannabinoid system, which include CB1 and CB2 receptors. Here we discuss the two receptors and their effects on inflammatory pain. To know a little bit of background info, it is important to understand where these receptors are found. CB1 receptors are predominantly found in the brain and CNS, while CB2 receptors are located outside the CNS (Figure 1) [6].

As the CB1 receptors predominantly lie in the CNS, there have been a variety of complications due to psychotomimetic and addiction (cannabinomimetic) adverse effects even when studies show selective CB1 agonists having analgesic effects [19]. This controversy affected the use of CB1 agonists, and therefore the majority of studies rely on the action of CB2 receptors in the mediation of inflammatory pain. The CB2 receptor-selective agonists have greatly displayed their action to reduce pain and inflammation, all while diminishing the unwanted behavioral effects cannabinoids are shown to have [7].

A cannabinoid agonist (HU-210) was studied for its anti-inflammatory and nociceptive effects on a rat. They concluded that CB1 receptors are involved in nociceptive pain and that both CB1 and CB2 receptors are involved in inflammatory hypersensitivity. The CB2 receptor worked by inhibiting inflammatory hypersensitivity thereby leading to anti-inflammation [20]. This can be therapeutic in inflammatory pain, especially in conditions such as osteoarthritis. Since CB2 receptors were mostly found outside the CNS, it suggested that it may have novel therapeutic properties to aid in inflammatory pain [6]. After a review of studies done on animal models, it was concluded that the findings were suggestive of CB2‐selective agonists demonstrating promising results in reducing inflammatory and neuropathic pain states [6]. The acute administration of the activation of the CB2 receptors showed positive results, but it is still unknown in a long-term treatment approach [6].

CB2-like receptors have been shown to have a role in modulating pain. The process indicates that CB2 receptors contained in peripheral immune tissue mediate analgesia by altering cytokine profiles [21]. Cytokines are inflammatory mediators that potentiate pain signals. Blunting the effect of cytokines may dampen the pain signals. An endogenous cannabinoid, anandamide, produces antinociception through mechanisms that differ from those of other types of cannabinoids. It acts on the vanilloid receptors but proves that the endocannabinoid system has physiological and/or pathophysiological roles in the modulation of pain [22]. The CB2 receptor studied in comparison with the CB1 receptor showed that the CB2 receptor plays a predominant role affecting joint pain and is likely to be involved in the adaptive changes in the opioids system, which was induced in the chronic pain state [23]. This beneficial aspect of controlling pain in the opioid system can lessen the burden of opioid side effects and complications.

The beneficial outcome of CB2 receptors showed that they reduced inflammatory nociception without affecting the overall behavior or CNS side effects [7]. A study was conducted on O-3223 (CB2 receptor-selective agonist), which showed some promising effects on chronic pain. The antihyperalgesic actions and control of nocieptive pain may be used to treat chronic inflammatory states [24]. Many studies promised that without the activation of the CB1 receptors, using a targeted approach on the selective CB2 receptors leads to great results clinically for the treatment of pain without CNS cannabinoid side effects [25].

Antinociceptive effects

The most commonly presented clinical complaint in the United States is chronic pain, which affects about 10% of the adult population [26]. The endocannabinoid system is composed of CB1, CB2 receptors, endogenous cannabinoid ligands, and enzymes, all of which are known to affect pain pathways [27]. Its antinociceptive effects are seen across acute, neuropathic, and inflammatory pain conditions. Through targeting the receptors and activating them, it has been an important part of treating and solving pain without the abuse of conventional treatment and opioid overuse. 

The focus of chronic inflammatory pain had been a significant area of interest as the antinociceptive effects may solve many chronic issues. The burden of chronic pain conditions usually requires a polypharmaceutical approach due to inflammatory as well as neuropathic pain. This is where physicians have turned to cannabinoids to treat these conditions to aid in the approach [28]. An example of an important chronic pain condition includes osteoarthritis (OA), which has been a burden of pain for patients with uncontrollable inflammation. Many patients in the United States have turned towards cannabinoids to treat OA pain, and there is a growing pool of studies that support its efficiency and effect on inflammatory pain [29].

Overall, antinociceptive effects studied in multiple trials showed that the major endocannabinoid system running in parallel with the opioid system could play a predominant role in the resolution of chronic pain states, including the affective and cognitive aspect of pain [18]. The inflammatory release of cytokines and the chronic burden of NSAID overuse can be redirected by targeting the cannabinoid receptors [18]. This lessens the side effects both by conventional treatment measures and producing dose-dependent antinociception through cannabinoid receptor targeting [18].

The antinociceptive effects of the cannabinoid system showed that the specific CB2 target approach led to little to no psychoactivity all while causing minimal side effects [30]. This approach leads to removing the social stigma against cannabis use and the potent psychotomimetic side effects and negative symptoms. These studies aimed at targeting the CB2 receptor showed more benefits in inflammatory pain states. A study done on a CB2 agonist (AM1241) showed that actions at CB2 receptors are sufficient to suppress inflammation-evoked neuronal activity to normalize nociceptive threshold while producing adequate antinociception in inflammatory pain states [31]. These promising results have been repetitively proving that inflammation and pain states can be solved with a targeted approach by the activation of the cannabinoid receptors, including cancer, spastic, neuropathic, acute, and chronic pain conditions (Table 1) [32].

**Table 1:** Mechanism of action OA, osteoarthritis; PPAR-α, peroxisome proliferator-activated receptor alpha

Author	Year of publication	Purpose of the study	Results/conclusions
Donvito et al. [3]	2017	Exploring the potential therapeutic approach for inflammatory and neuropathic pain conditions using preclinical and clinical evidence of various endocannabinoid systems	Diverse pain states were consistently controlled using the cannabis and cannabinoid receptor agonists
Richardson [4]	2000	To determine if cannabinoids produce antinociception and antihyperalgesic by acting at peripheral, spinal, and supraspinal sites to inhibit mast cell degranulation, primary afferent activity, and responses of nociceptive neurons	The use of peripherally selective cannabinoid targets showed enhancement of pain relief, especially with co-administration of alternative options including morphine, and showed therapeutic effects while minimizing unwanted side effects.
Klien [5]	2005	To study the use of specific drugs in the treatment of chronic inflammatory conditions	Showed that these drugs also have immunosuppressive and anti-inflammatory properties
Demuth and Molleman [10]	2005	To determine the exact cellular signaling mechanisms of cannabinoid receptors and their interaction within the human body	Concluded that multiple G protein-coupled receptors played a role in cannabinoid receptor cellular signaling, including proving that cannabinoids have alternative targets other than the CB receptors.
Xiong et al. [15]	2011	To study the cannabinoid-GlyR interaction	Concluded that the mice without the a3GlyRs had no analgesic effects on the CB1 and CB2 receptors. Mice require the potentiation of the cannabinoid-Gly interaction to contribute to cannabinoid-induced analgesia and therapeutic benefits.
Alsalem et al. [12]	2019	To study the synthetic cannabinoids using rat models of OA and assess the relation of the CB1 receptor and the PPAR-α receptor in mediating these effects	Both CB1 and PPAR-α receptors are involved in affecting pain in osteoarthritis. Targeting of these receptors promised great results in therapeutic effects.
Milligan et al. [1]	2020	To study the CB1 receptor and determine its role in analgesic properties	The nociceptive properties of the CB1 were analysed.
Grenald et al. [18]	2017	Using rodent models of acute and chronic inflammatory pain, it was studied if there was a relationship between morphine and CB2 agonist (JWH015) in treating these conditions.	It was concluded that the co-administration of morphine with JWH015 synergistically inhibited preclinical inflammatory, post-operative, and neuropathic-pain in a dose- and time-dependent manner.
Mackie [8]	2006	To study the targets of the cannabinoid receptors	Cannabinoid receptors can be activated or inhibited by agonists and antagonists, respectively. The G protein-coupled receptors play an important role in regulating a variety of human functions, including pain.

Limitations

The studies overall showed positive results, but had potential limitations. They were subjected to certain biases. Firstly, most of the animal study models could have a subjective estimation of pain and antinociceptive effects. As pain is subjective, it is difficult to measure an exact effect in the treatments of chronic conditions. Secondly, the research was limited by the measures used to perform these studies. Thirdly, these studies were not able to see if the cannabinoid receptors were directly part of the pain pathway, or if affected pain signals directly in the thalamus. All these limitations will need to be further investigated to have a greater understanding of the cannabinoid receptors and their relation to chronic pain.

## Conclusions

The understanding of the mechanism of action, types of different cannabinoid receptors and the antinociceptive effects in chronic inflammatory pain have played an important role in understanding how they can revolutionize medical advances in chronic pain control. Whether they reduce the burden of opioid use or decrease the excessive use of analgesics, their positive manifestations have shown to alleviate symptoms in a variety of chronic inflammatory conditions. Though their exact role in the immune system is unclear, they have been shown to reduce the burden of pain in chronic states.

On the contrary, the use of cannabis has many side effects, specifically psychotropic manifestations. This problem has been overcome by agonistic activity specifically on the CB2 receptor to minimize CNS activation. Further advances will be required to formulate medical drugs that work strictly on CB2 receptors distinctly. It is also required to investigate further safer routes of ingestion of these drugs rather than smoking, as it is more harmful rather than therapeutic to chronic users. In summary, it can widely benefit to reduce the burden of opioid overuse and side effects from conventional drugs, with the ultimate goal of relieving the pain that most of the patients suffer from on a daily basis.
